# MicroRNA signature for estimating the survival time in patients with bladder urothelial carcinoma

**DOI:** 10.1038/s41598-022-08082-7

**Published:** 2022-03-09

**Authors:** Srinivasulu Yerukala Sathipati, Ming-Ju Tsai, Sanjay K. Shukla, Shinn-Ying Ho, Yi Liu, Afshin Beheshti

**Affiliations:** 1 Center for Precision Medicine Research, Marshfield Clinic Research Institute, Marshfield, WI 54449 USA; 2 Hinda and Arthur Marcus Institute for Aging Research at Hebrew Senior Life, Boston, MA USA; 3 Department of Medicine, Beth Israel Deaconess Medical Center and Harvard Medical School, Boston, MA USA; 4 Institute of Bioinformatics and Systems Biology, National Yang Ming Chiao Tung University, Hsinchu, Taiwan; 5 College of Health Sciences, Kaohsiung Medical University, Kaohsiung, Taiwan; 6 Biomedical Engineering, National Yang Ming Chiao Tung University, Hsinchu, Taiwan; 7 KBR, Space Biosciences Division, NASA Ames Research Center, Moffett Field, CA 94035 USA; 8 Stanley Center for Psychiatric Research, Broad Institute of MIT and Harvard, Cambridge, MA 02142 USA

**Keywords:** Computational biology and bioinformatics, Cancer genomics

## Abstract

Bladder urothelial carcinoma (BLC) is one of the most common cancers in men, and its heterogeneity challenges the treatment to cure this disease. Recently, microRNAs (miRNAs) gained promising attention as biomarkers due to their potential roles in cancer biology. Identifying survival-associated miRNAs may help identify targets for therapeutic interventions in BLC. This work aims to identify a miRNA signature that could estimate the survival in patients with BLC. We developed a survival estimation method called BLC-SVR based on support vector regression incorporated with an optimal feature selection algorithm to select a robust set of miRNAs as a signature to estimate the survival in patients with BLC. BLC-SVR identified a miRNA signature consisting of 29 miRNAs and obtained a mean squared correlation coefficient and mean absolute error of 0.79 ± 0.02 and 0.52 ± 0.32 year between actual and estimated survival times, respectively. The prediction performance of BLC-SVR had a better estimation capability than other standard regression methods. In the identified miRNA signature, 14 miRNAs, hsa-miR-432-5p, hsa-let-7e-3p, hsa-miR-652-3p, hsa-miR-629-5p, and hsa-miR-203a-3p, hsa-miR-129-5p, hsa-miR-769-3p, hsa-miR-570-3p, hsa-miR-320c, hsa-miR-642a-5p, hsa-miR-496, hsa-miR-5480-3p, hsa-miR-221-5p, and hsa-miR-7-1-3p, were found to be good biomarkers for BLC diagnosis; and the six miRNAs, hsa-miR-652-5p, hsa-miR-193b-5p, hsa-miR-129-5p, hsa-miR-143-5p, hsa-miR-496, and hsa-miR-7-1-3p, were found to be good biomarkers of prognosis. Further bioinformatics analysis of this miRNA signature demonstrated its importance in various biological pathways and gene ontology annotation. The identified miRNA signature would further help in understanding of BLC diagnosis and prognosis in the development of novel miRNA-target based therapeutics in BLC.

## Introduction

Bladder urothelial carcinoma (BLC) is one of the major causes of cancer moralities with nearly 17,200 deaths and 83,730 estimated new cases in 2021 in United States alone; and 549,000 estimated new cases and 200,000 deaths globally^1,2^. According to estimates, 440,864 cases in men and 132,414 cases in women have been reported in 2020^1^. A male predominance is observed in all BLC cases which was ranked as the 6th most common cancer and 9th leading causes of cancer among men globally^1^. The risk factors of BLC include occupational exposure to carcinogenic substances and cigarette smoking which is considered as the major risk factor in both genders and accounts for 47% of all these cases^3,4^. BLC presents in two different forms, non-muscle-invasive tumors (NMIBC) and muscle-invasive tumors (MIBC). The NMIBC is benign with a higher incidence rate whereas MIBC is aggressive, could metastasize but lower incidence^5^. The standard treatment includes the combination of cytology and cystoscopy for the prognosis and diagnosis of BLC. There are some outstanding issues remaining in treatment conditions such as poor sensitivity of cytology in tumor detection, and invasiveness of cystoscopy^6^. Additionally, difference exists in response to similar treatment among patients because tumor heterogeneity makes it challenging to cure cancer and therapeutic modalities greatly affects the quality of life in elderly patients^7^. The five-year survival rate for patients with MIBC is 45%, and lymph node metastasis causes poor survival of 5% regardless of type of the treatment^8,9^. Although considerable advancements in adjuvant chemotherapy and surgery, BLC continues to be a common cancer. Therefore, identifying the survival related variants that could contribute to development of novel therapeutic strategies is necessary to improve the survival in patients with BLC.

MicroRNAs (miRNAs) are small, endogenous, non-coding RNAs involved in translation repression by posttranscriptional regulation of gene expression^10^. MiRNAs have been implicated in cancer progression and they can either promote or suppress tumor progression and metastasis^11–13^. A growing body of evidences has shown the association of miRNAs with cancer progression, diagnosis, and prognosis^14,15^, especially that of BLC^16,17^. For instance, significant changes in miRNA expression were observed in clinical samples, and three miRNAs, miR-129, miR-133b, and miR-518c, were discovered as potential prognostic predictors associated with BLC progression^18^. Li et al. identified urothelial carcinoma associated miRNAs that were significantly expressed in urine and plasma samples of patients with chronic kidney disease^19^. Ichimi et al. identified seven miRNAs namely, miR-145, miR-30a-3p, miR-133a, miR-133b, miR-195, miR-125b, and miR-199a, which were significantly downregulated in BLC when compared to the normal samples^20^. Lin and colleagues used hybridization-based miRNA array on BLC and found that miR-143 functioning as a tumor suppressor, and 14 down-regulated miRNAs that were significantly expressed between tumor and normal samples^21^. Studies have also noted the aberrant expression of miRNAs in tumor compared to normal bladder tissues^22–25^. However, there are limited studies on estimating survival using machine learning techniques to explore the roles of miRNAs in terms of survival association in BLC.

**Figure 1:**
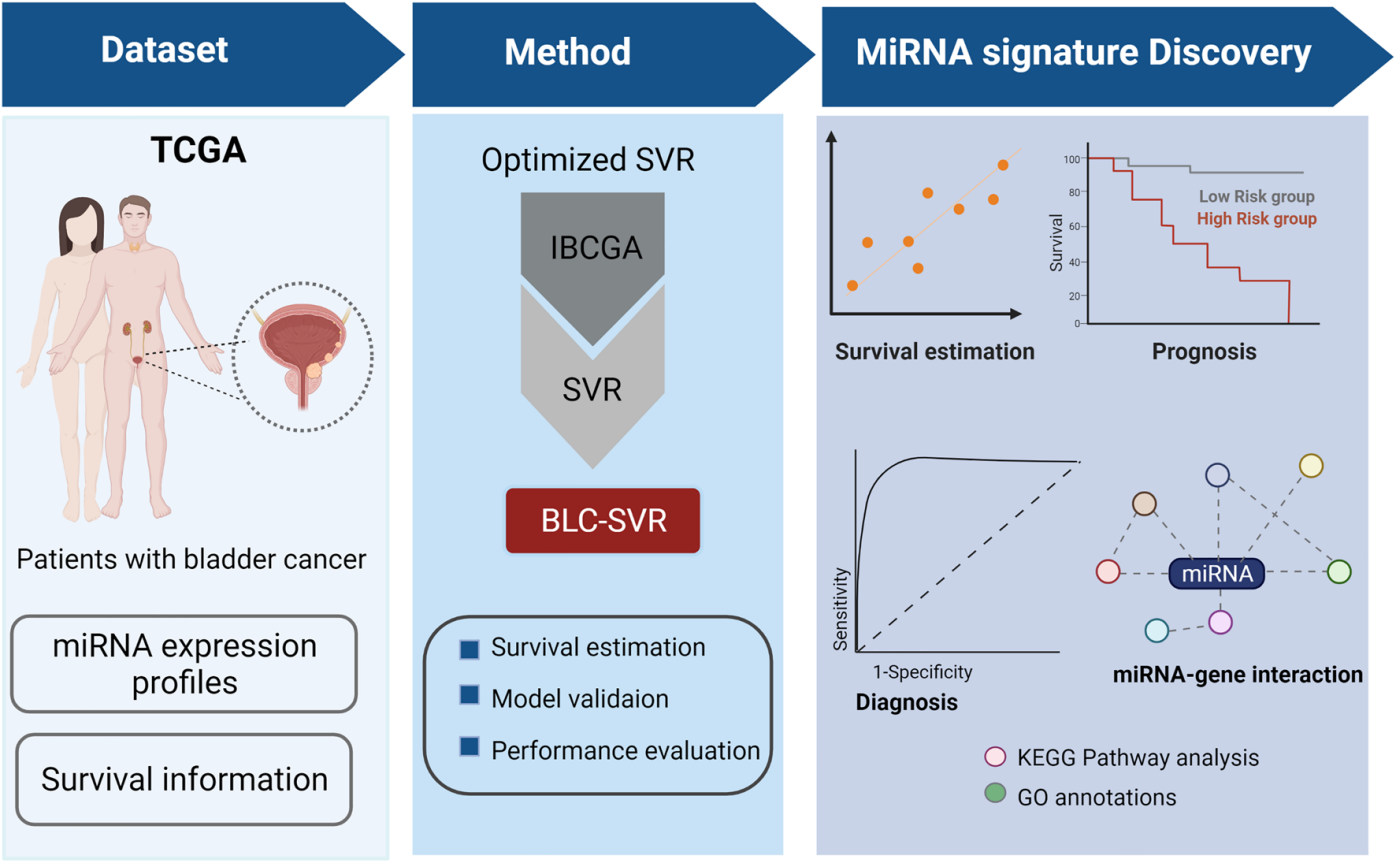
System flowchart of BLC-SVR. miRNA expression profiles of BLC patients with survival information were used as input data of the BLC-SVR method, and the outputs were the miRNA signature with the predicted survival time.

Previously, we identified miRNA signatures for predicting the cancer stages in breast and hepatocellular carcinoma^26,27^, and estimating the survival in glioblastoma, lung adenocarcinoma, and ovarian cancers^28–31^. In this study, we developed a survival time estimator called BLC-SVR to estimate the survival in BLC patients using miRNA expression profiles. BLC-SVR was developed based on support vector regression (SVR) incorporating with an optimal feature selection algorithm IBCGA^32^ to identify the survival associated miRNA signature and estimate the survival in patients with BLC. BLC-SVR achieved a promising accuracy on estimating the survival time of patients with BLC. Furthermore, bioinformatics analysis on the identified miRNAs to explore their diagnostic and prognostic abilities in BLC. The overview of the BLC-SVR is shown in Fig. 1.

## Results

### Identification of miRNA signature for estimating survival time

We retrieved 106 miRNA expression profiles of patients with BLC from The Cancer Genome Atlas (TCGA) database. Each miRNA profile consisted of 485 miRNAs which were the variables for survival estimation. BLC-SVR identified a set of miRNAs as a signature for estimating the survival time in patients with BLC. A robust miRNA signature was selected by performing 50 independent runs of BLC-SVR. The appearance score (*ASC*) for each miRNA signature of the prediction model was measured and scored according to their frequency among independent runs. The miRNA signature with a highest *ASC* accommodates the more frequent miRNAs among the independent runs of BLC-SVR. The average and highest *ASCs* obtained from 50 independent runs were 13.52 ± 1.60, and 17.27, respectively. The robust signature with the highest *ASC* consisted of 29 miRNAs and obtained a squared correlation coefficient (R^2^) and mean absolute error (MAE) of 0.81 and 0.51 year, between actual and estimated survival times, respectively. The diagnostic and prognostic prediction ability of the identified miRNA signature is discussed in the following sections. The *ASCs* for all the independent runs of BLC-SVR are depicted in Supplementary Fig. S1.

### Prediction performance comparison

We compared the prediction performance of BLC-SVR with standard machine learning methods, including ridge regression, least absolute shrinkage selection operator (Lasso), and elastic net. Ridge regression achieved a R^2^ and MAE of 0.42 and 0.81 year, between actual and estimated survival times, respectively; Lasso obtained a R^2^ and MAE of 0.50 and 0.74 year, between actual and estimated survival times, respectively; and elastic net obtained a R^2^ and MAE of 0.52 and 0.73 year, between actual and estimated survival times, respectively. BLC-SVR obtained a mean performance of 50 runs (BLC-SVR-Mean) with R^2^ and MAE of 0.79 ± 0.02 and 0.52 ± 0.32 year, between actual and estimated survival times, respectively. Whereas the best performance (BLC-SVR-Best) has the largest R^2^ and MAE of 0.83 and 0.516 year using 32 miRNAs, respectively. The prediction comparison results showed the better estimation capability of BLC-SVR than the popular regression methods. The comparison of prediction performance of BLC-SVR with some regression methods is shown in Table 1. The correlation plots of BLC-SVR, ridge regression, Lasso, and elastic net are shown in Supplementary Fig. S2.

**Table 1:** The comparison of prediction performance.

Method	R^2^	MAE (years)	Features selected
Ridge regression	0.42	0.81	485
Lasso	0.50	0.74	26
Elastic net	0.52	0.73	33
BLC-SVR-ASC	0.81	0.51	29
BLC-SVR-Best	0.83	0.51	32
BLC-SVR-Mean	0.79 ± 0.02	0.63 ± 0.32	32.64 ± 4.22

R^2^, squared correlation coefficient; MAE, mean absolute error.

Next, we validated the estimation ability of BLC-SVR using an independent test cohort from the TCGA database. The independent test cohort consisting of 123 patients with BLC along with their follow-up times up to one year with an average 5.54 months. BLC-SVR estimated mean survival time of these patients was 15.50 months. There were 93 patients whose predicted survival time was longer than the actual follow-up time. The estimation performance of BLC-SVR achieved 75.60% accuracy on estimating the patients’ survival time. However, for the remaining 30 patients, estimated survival time was slightly shorter than the follow-up time. The prediction performance of BLC-SVR on 123 patients is shown in Supplementary Fig. S3.

Next, we prioritized the miRNAs of the signature using main effect difference (MED) analysis based on their contribution to the estimation of survival time as described in the study^33^. The top 10 ranked miRNAs of the signature, including hsa-miR-432-5p, hsa-let-7e-3p, hsa-miR-146b-5p, hsa-miR-505-3p, hsa-miR-652-3p, hsa-miR-629-5p, hsa-miR-193b-5p, hsa-miR-203a-3p, hsa-miR-542-5p, and hsa-miR-128-3p, were analyzed further. The miRNAs signature and their corresponding MED scores and ranks are listed in Table 2.

**Table 2:** ROC analysis of top ranked miRNAs between tumor and normal samples.

RANK	miRNA	MIMAT_ID	MED	AUC
1	hsa-miR-432-5p	MIMAT0002814	1.53	0.81
2	hsa-let-7e-3p	MIMAT0004485	1.50	0.81
3	hsa-miR-146b-5p	MIMAT0002809	1.47	0.66
4	hsa-miR-505-3p	MIMAT0002876	1.34	0.59
5	hsa-miR-652-3p	MIMAT0003322	1.12	0.82
6	hsa-miR-629-5p	MIMAT0004810	0.96	0.81
7	hsa-miR-193b-5p	MIMAT0004767	0.95	0.64
8	hsa-miR-203a-3p	MIMAT0000264	0.80	0.71
9	hsa-miR-542-5p	MIMAT0003340	0.79	0.53
10	hsa-miR-128-3p	MIMAT0000424	0.79	0.67
11	hsa-miR-129-5p	MIMAT0000242	0.66	0.85
12	hsa-miR-769-3p	MIMAT0003887	0.64	0.79
13	hsa-miR-224-3p	MIMAT0009198	0.57	0.59
14	hsa-miR-570-3p	MIMAT0003235	0.56	0.77
15	hsa-miR-1254	MIMAT0005905	0.54	–
16	hsa-miR-143-5p	MIMAT0004599	0.53	0.67
17	hsa-miR-320c	MIMAT0005793	0.43	0.7
18	hsa-miR-642a-5p	MIMAT0003312	0.38	0.74
19	hsa-miR-496	MIMAT0002818	0.35	0.7
20	hsa-miR-421	MIMAT0003339	0.35	0.5
21	hsa-miR-2116-3p	MIMAT0011161	0.31	0.59
22	hsa-miR-361-5p	MIMAT0000703	0.29	0.66
23	hsa-miR-548o-3p	MIMAT0005919	0.26	0.73
24	hsa-miR-26a-1-3p	MIMAT0004499	0.16	0.64
25	hsa-miR-339-3p	MIMAT0004702	0.08	0.49
26	hsa-miR-23a-5p	MIMAT0004496	0.07	0.63
27	hsa-miR-508-3p	MIMAT0002880	0.06	0.58
28	hsa-miR-221-5p	MIMAT0004568	0.02	0.72
29	hsa-miR-7-1-3p	MIMAT0004553	0.012	0.71

MED, main effect difference; AUC, area under the receiver operating curve.

### The roles of top 10 ranked miRNAs in cancer

The literature validation on top ranked miRNAs revealed that these miRNAs possess different functions and active involvement in BLC progression (Table 3). For instance, the up-regulated hsa-miR-432-5p targets RNA-binding motif protein 5 and regulate apoptosis in bladder cancer cells^34^. Hsa-let-7 family is known to be differentially expressed in various cancers including BLC^35,36^. Hsa-miR-146b expression was upregulated in bladder cancer tissues when compared to the normal tissues^37^. A real-time quantitative polymerase chain reaction (RT-qPCR) study on BLC cell lines reported that hsa-miR-652-3p expression levels were upregulated and knockdown of this miRNA significantly affected cell proliferation, migration, and invasion in BLC^38^. A PCR based miRNA screening study revealed that hsa-miR-193 downregulated the expression of oncogenes, Cyclin D1 and *EST1*, and inhibited cell migration in human urothelial cells^39^. The hsa-miR-203a has been identified as a tumor suppressor and its overexpression inhibits cell proliferation, invasion and migration in BLC^40^. Hsa-miR-542 expression was downregulated and negatively correlated with the expression of *surviving* protein resulting in the inhibition of proliferation in BLC cells^41^.

**Table 3:** The summary of the roles of miRNAs in bladder cancer.

miRNA	Regulation	Functional impact on tumors	Target gene for bladder cancer	References
hsa-miR-432-5p	Up	Tumor progression	RBM5	^34^
hsa-let-7e-3p	Up	Tumor progression	LIN28, HMGA2, MYC, BCL2L	^35,72^
hsa-miR-146b-5p	Up	Tumor progression	ETS2	^37^
hsa-miR-505-3p	N/A	N/A	N/A	N/A
hsa-miR-652-3p	Up	Tumor progression	KCNN3	^38^
hsa-miR-629-5p	N/A	N/A	N/A	N/A
hsa-miR-193b-5p	down	Tumor suppression	CCND1 and ETS2	^39^
hsa-miR-203a-3p	Down	Tumor suppression	SIX4	^40^
hsa-miR-542-5p	Down	Tumor suppression	BIRC5	^41^
hsa-miR-128-3p	N/A	N/A	N/A	N/A

N/A, not available.

The roles of three of the top 10 ranked miRNAs (hsa-miR-505-3p, hsa-miR-629-5p, and hsa-miR-128-3p) have not been previously reported in BLC. However, these miRNAs are implicated in other major cancers. For instance, hsa-miR-505-3p acts as a tumor suppressor in pancreatic cancer and hepatocellular carcinoma^42,43^. Hsa-miR-629-5p promotes tumor progression by targeting *AKAP13* in prostate cancer^44^, and hsa-miR-128-3p acts as tumor suppressor in breast cancer by regulating the *LIMK1*/*CFL1* signaling pathway^45^. Hence, their involvement in other major cancers suggests that their expression is biologically consistent and important in BLC. A summary of miRNAs and their regulation in BLC is shown in Table 3.

### Diagnostic ability of the miRNAs

**Figure 2:**
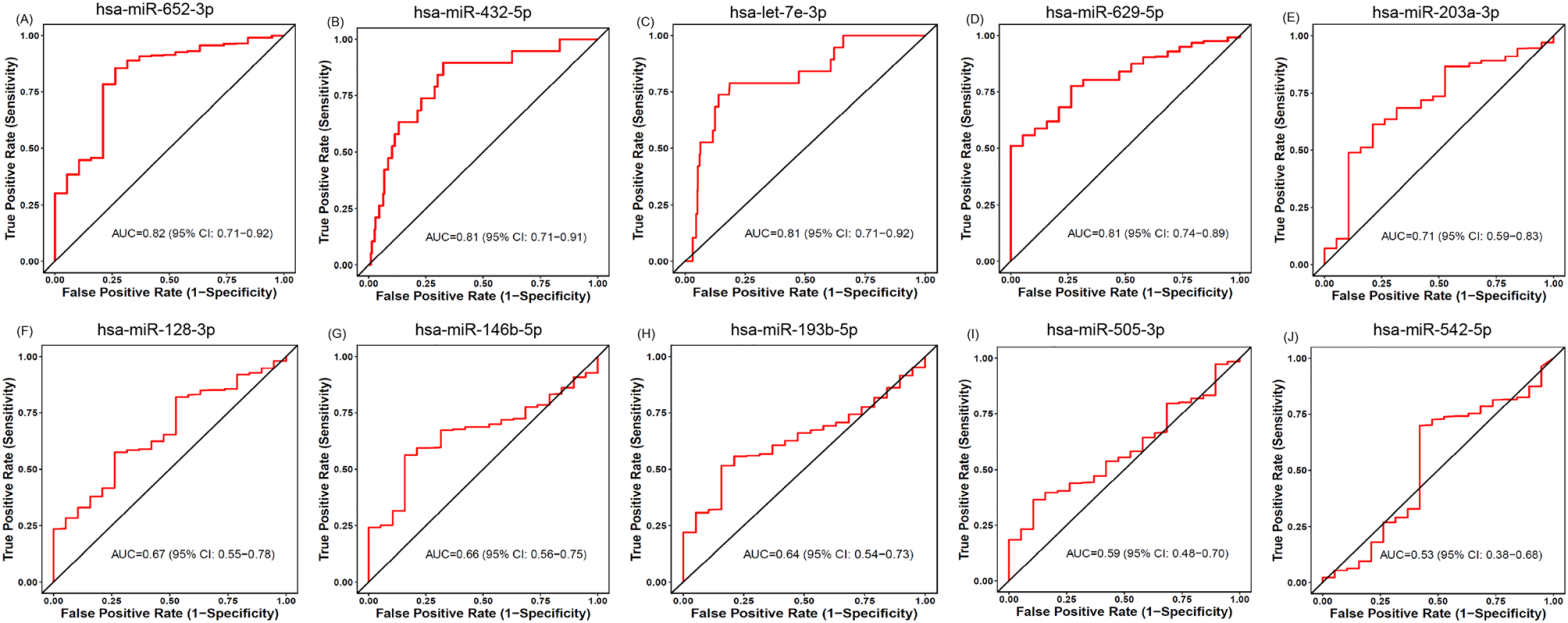
ROC curve analysis of the top 10 ranked miRNAs. (**A**) hsa-miR-652-3p, (**B**) hsa-miR-432-5p, (**C**) hsa-let-7e-3p, (**D**) hsa-miR-629-5p, (**E**) hsa-miR-203a-3p, (**F**) hsa-miR-128-3p, (**G**) hsa-miR-146b-5p, (**H**) hsa-miR-193b-5p, (**I**) hsa-miR-505-3p, and (**J**) hsa-miR-542-5p.

To determine the diagnostic ability of the identified miRNA signature, receiver operating curve (ROC) analysis was performed using BLC tumor and normal samples. The ROC analysis showed that 14 miRNAs of the signature have the good diagnostic ability (AUC ≥ 0.7) while distinguishing the tumor and normal samples, shown in Table 2. The top 10 ranked miRNAs obtained an average area under the ROC curve (AUC) of 0.70 ± 0.10 and five of these miRNAs showed good diagnostic ability. The five miRNAs, hsa-miR-432-5p, hsa-let-7e-3p, hsa-miR-652-3p, hsa-miR-629-5p, and hsa-miR-203a-3p obtained AUCs of 0.81, 0.81, 0.82, 0.81, and 0.71, respectively. The ROC curves for the top 10 ranked miRNAs are shown in Fig. 2. Further, we combined the top 10 ranked miRNAs to predict the diagnosis of BLC using Random forest classifier^46^. We used a dataset consisting of 418 tumor samples and 18 normal samples retrieved from the TCGA. The prediction model was selected after 100 iterations of 10-CV. Random forest obtained a 10-CV accuracy, sensitivity, specificity, and AUC of 96.02%, 0.96, 0.85, and 0.95, respectively, while distinguishing tumor and normal samples, shown in Supplementary Fig. S4. The combination of top 10 ranked miRNAs showed better diagnostic ability.

**Figure 3:**
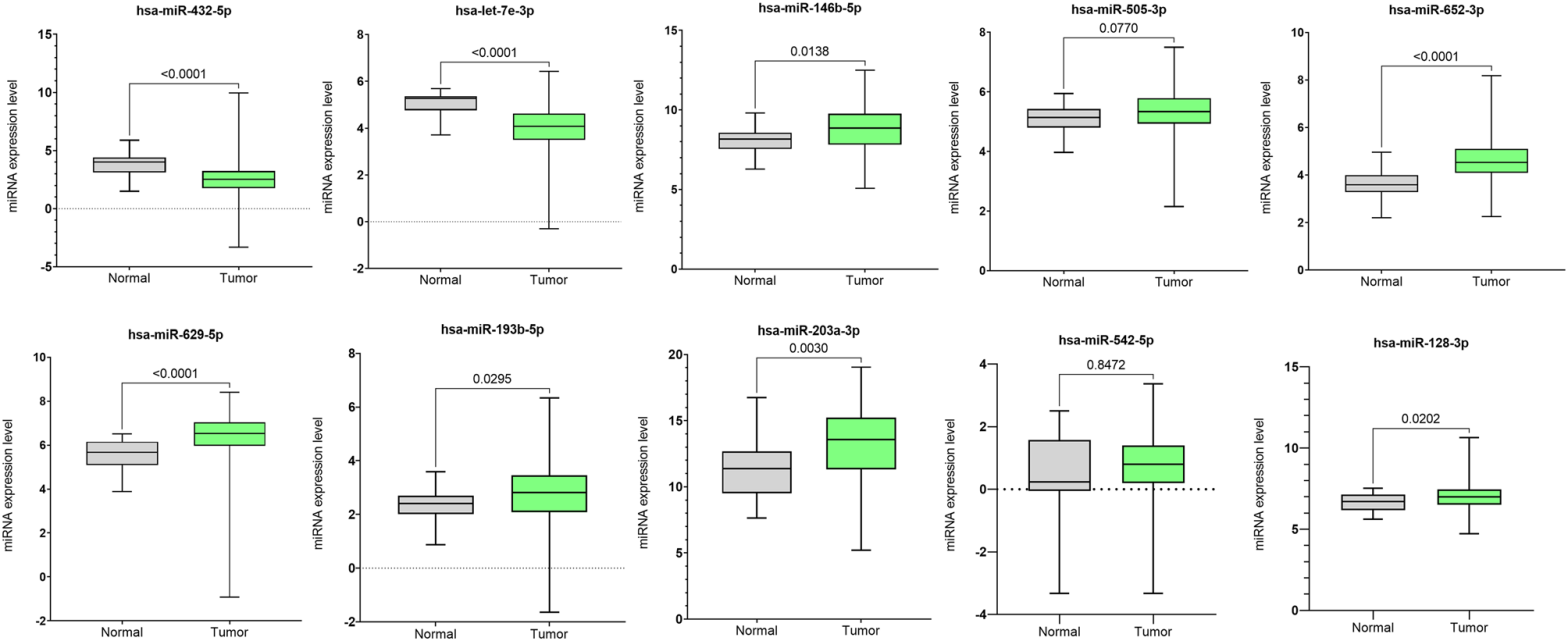
Expression differences of the top 10 ranked miRNAs between tumor and normal samples using box plots.

Additionally, expression differences of the top 10 ranked miRNAs between normal and tumor samples were analyzed using box-plot analysis. The analysis showed that eight miRNAs, hsa-miR-432-5p, hsa-let-7e-3p, hsa-miR-146b-5p, hsa-miR-652-3p, hsa-miR-629-5p, hsa-miR-193b-5p, hsa-miR-203a-3p, and hsa-miR-128-3p were significantly expressed (p < 0.05) between normal and tumor samples. The box plot analysis of the top 10 ranked miRNAs is shown in Fig. 3.

### Prognostic ability of the miRNAs

**Figure 4:**
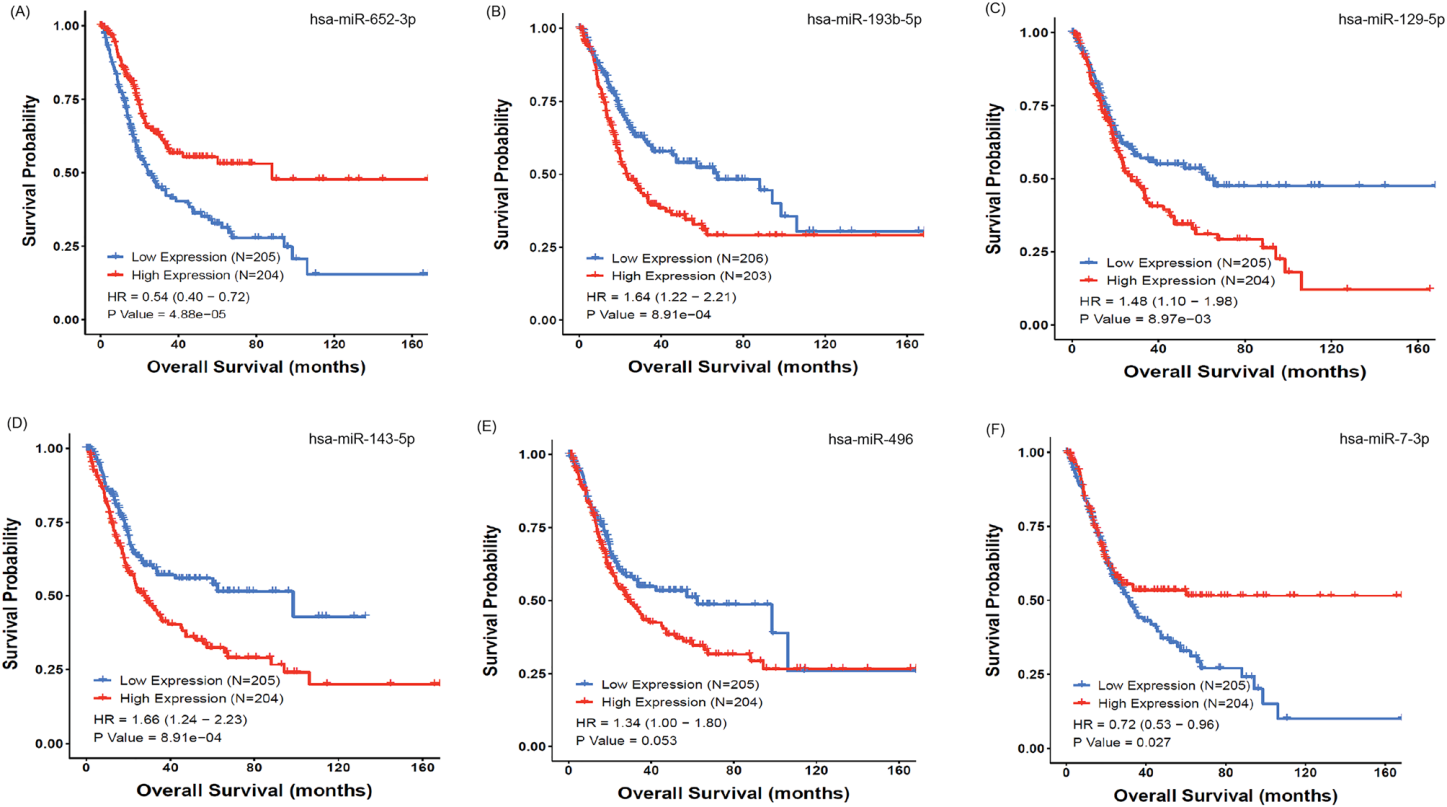
Kaplan-Meir survival analysis. The survival probability of patients with high expression of miRNAs (**A**–**D**) is poorer than that of patients with low expression. The high expression of hsa-miR-652-5p and hsa-miR-7-3p (**E**, **F**) has positive effect on survival probability.

The prognostic performance of miRNA signature was analyzed by Kaplan-Meir (KM) survival curves using CancerMIRNome^47^. Six miRNAs of the signature showed significant prognosis capability in overall survival analysis. These six miRNAs, hsa-miR-652-5p, hsa-miR-193b-5p, hsa-miR-129-5p, hsa-miR-143-5p, hsa-miR-496, and hsa-miR-7-1-3p, obtained p-values of 4.88e−05, 8.91e−04, 8.97e−03, 8.91e−04, 0.05, and 0.027, respectively, between high and low expression groups. The KM survival curves for the six miRNAs are shown in Fig. 4.

### Biological significance of the miRNA signature

To determine the biological relevance of the miRNA signature that could aid in understanding the functional information and involvement in disease-associated pathways, Kyoto Encyclopedia of Genes and Genomes (KEGG) and Gene Ontology (GO) analysis were employed. The miRNA signature is significantly involved in various biological pathways such as prion disease, fatty acid biosynthesis, fatty acid metabolism, ECM-receptor interaction, hippo signaling pathway, adherence junction, steroid biosynthesis, lysine degradation, TGF-beta signaling pathway, and proteoglycans in cancer. The number of targeted genes involved in KEGG pathways of the miRNA signature is shown in supplementary Table S1. The miRNA signature enriched in KEGG pathways is shown in Supplementary Fig. S5.

Next, the biological significance of the miRNA signature was analyzed in different stages of BLC using KEGG pathway analysis. The differentially expressed miRNAs of the signature were identified between stage II, III, and IV. There were nine miRNAs, including hsa-miR-496, hsa-miR-146b-5p, hsa-miR-652-3p, hsa-miR-26a-1-3p, hsa-miR-193b-5p, hsa-miR-642a-5p, hsa-miR-432-5p, hsa-miR-143-5p, and hsa-miR-505-3p which were differentially expressed between stage II&III, and two miRNAs, hsa-miR-143-5p and hsa-let-7e-3p, between stage III&IV. The significant biological pathways in stage II&III were thyroid hormone synthesis, oxytocin signaling pathway, ErbB signaling pathway, long-term depression, and hippo signaling pathway, to name a few. The significant pathways in stage III&IV were biosynthesis of unsaturated fatty acids, ErbB signaling pathway, GABAergic synapse, morphine addiction, and estrogen signaling pathway. There were some common pathways, including ErbB signaling pathway, thyroid hormone signaling, morphine addiction, non-small cell lung cancer, and estrogen signaling pathway in stage II&III and stage III&IV. However, some targeted pathways were different across cancer stages of BLC. The complete list of significant pathways across cancer stages are listed in Supplementary Table S2. The bubble plots showing the KEGG pathways in BLC stages are shown in Supplementary Figs. S6&S7.

Next, GO annotations of the miRNA signature was employed in three categories, including biological process, molecular functions, and cellular components. The GO analysis showed that the miRNA signature involved several biological processes that the top five significant biological processes were DNA metabolic process, cellular protein metabolic process, membrane organization, RNA metabolic process, and nucleobase-containing compound catabolic process (Supplementary Table S3). The top five molecular functions were ion binding, nucleic acid binding transcription factor activity, protein binding transcription factor activity, enzyme binding, and enzyme regulatory activity. The top five cellular components were organelle, cytosol, nucleoplasm, protein complex, and focal adhesion. The details of GO annotations for the miRNA signature are listed in Supplementary Tables S3-S5. The GO enrichment analysis of the miRNA signature is depicted in Supplementary Figs. S8-S10.

### Gene interaction network

**Figure 5:**
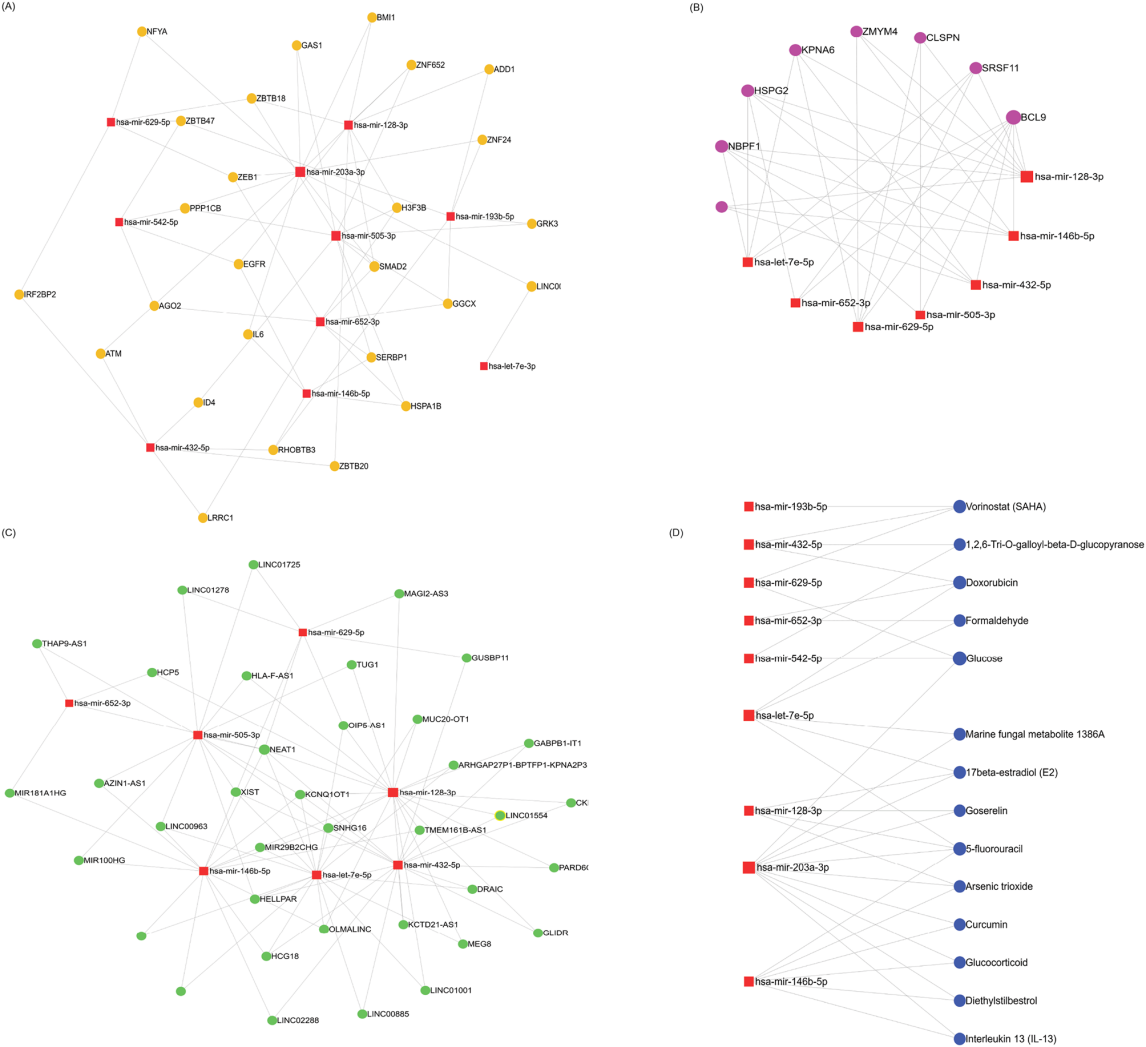
MiRNA-interaction network. (**A**) miRNA-gene interaction network, (**B**) miRNA-ciRNA interaction network, (**C**) miRNA-lncRNA interaction network, and (**D**) miRNA-small molecule interaction network.

The complex networks in which miRNAs engaged with other functional molecules can influence cell biological responses and human diseases^48^. Hence, a miRNA network analysis was employed for the top 10 ranked miRNAs with genes, long non-coding RNAs (lncRNAs), circular RNAs (ciRNAs), and small molecules to explore the miRNA-target interactions using miRNet 2.0: a miRNA-centric network visual analytics platform^49^. The miRNA-gene target interaction network was built with experimentally validated gene target networks using the miRTarBase V8.0^50^. There were 1594 gene interactions with top 10 ranked miRNAs in a miRNA-gene target network. The miRNA-gene target network is shown in Fig. 5A.

In the miRNA-lncRNA interaction network, seven of the top 10 ranked miRNAs targeted 197 lncRNAs formed with 255 edges. There were 4508 circular RNAs (ciRNAs) formed with 7751 edges in the miRNA-ciRNA interaction network. In the miRNA-small molecule interaction network, there were 43 compounds interacting with top 10 ranked miRNAs and forming 65 edges. The miRNA interaction networks for genes, lncRNA, ciRNA, and small molecules are shown in Fig. 5A–D.

## Discussion

The critical role of miRNAs in cancer biology has opened up a new direction for oncology research. Numerous evidences have demonstrated the development of miRNA-based cancer progression, diagnosis, and therapeutics^51–53^. Bladder cancer is one of the common cancers and a heterogeneous disease with prognostic and therapeutic challenges. Identifying the survival related variants could help understand the cancer survival at various stages and may contribute to the therapeutic improvements in BLC. Due to cost and time consumption in experimental methods to predict the targets and identify biomarkers, computational methods are often used in miRNA biology and cancer prognosis predictions. Advances in machine learning methods have significant importance in developing fast and accurate models to aid in caner prognosis, diagnosis, and medical decision-making^54^. Recent developments on miRNA-disease associations revealed the importance of computational models in understanding the disease associated variants^55^. However, there are limited studies on identifying miRNA signatures to estimate the survival time in patients with BLC using machine learning techniques.

The machine learning methods often suffer from higher dimensionality issues^56^, especially in biomedical data. The used feature selection algorithms could work well in coping with the curse of dimensionality issue resulting from genomic data and select a robust signature for cancer prognosis^31,57^. To address the dimensionality issue, we used an optimal feature selection algorithm IBCGA to identify a small set of miRNAs from a large number of candidate miRNAs that are associated with survival time in patients with BLC. In our previous studies, optimized survival estimation methods were developed to estimate the survival time in patients with glioblastoma, lung adenocarcinoma, and ovarian cancers^28–30^. In this study, we developed an optimized SVR-based method BLC-SVR to identify a miRNA signature associated with survival and estimate the survival time in patients with BLC. The identified miRNA signature consisted of 29 miRNAs as a signature and obtained a R^2^ and MAE of 0.81 and 0.51 year, between actual and predicted survival times, respectively. The estimation capability of BLC-SVR was compared with some standard regression methods and results showed its promising estimation performance. Further, the identified miRNAs of the signature were ranked based on their contribution to the estimation performance. The literature survey on top 10 ranked miRNAs demonstrated that seven of top 10 ranked miRNAs are actively involved in BLC progression except the three miRNAs hsa-miR-505-3p, hsa-miR-629-5p, and hsa-miR-128-3p. These three miRNAs are important contributors to the estimated performance. Therefore, hsa-miR-505-3p, hsa-miR-629-5p, and hsa-miR-128-3p may be novel targets for BLC and further studies are needed to validate their roles in BLC.

In addition, the diagnostic ability prediction results showed that five of the top 10 ranked miRNAs, hsa-miR-432-5p, hsa-let-7e-3p, hsa-miR-652-3p, hsa-miR-629-5p, and hsa-miR-203a-3p, obtained an AUC greater than 0.70 while distinguishing the tumor and normal samples, proving their discrimination ability. The differential expression analysis on top 10 ranked miRNAs showed that eight of the top 10 ranked miRNAs were significantly expressed between tumor and normal samples. Next, KM survival analysis of the miRNA signature revealed that six miRNAs, hsa-miR-652-5p, hsa-miR-193b-5p, hsa-miR-129-5p, hsa-miR-143-5p, hsa-miR-496, and hsa-miR-7-1-3p, were good prognostic predictors of overall survival in patients with BLC.

The functional analysis of miRNAs revealed the involvement of miRNAs in physiological process that are essential for disease mechanism. Biological relevance of the identified miRNA signature concluded that the miRNA signature was involved in several biological pathways, including biological processes, molecular functions, and cellular components. The top-3 KEGG pathways were prion diseases, fatty acid biosynthesis, and fatty acid metabolism. In human prion diseases, point mutations in the prion protein gene (*PRNP*), which encodes *PrP*, induce familial forms of human prion diseases^58^. Somatic missense mutation in the prion protein gene (*PRNP*) have been identified in patients with BLC^59^. Urinary retention as an early symptom was observed in patients with prion disease^60^. The prion proteins were detected in urine and involved in disease infection^61^. Fatty acid synthesis and fatty acid metabolism pathways are associated with various cancers including BLC^62^. A previous study showed that the change in the fatty acid composition may be an indicator of altered lipid metabolism occurring in vivo during human bladder tumorigenesis. The bladder cancer tissue showed a significant reduction in total n-6 polyunsaturated fatty acid (− 15.1%; P < 0.001)^63^.

The pathway analysis demonstrated that the group of miRNAs target specific pathways and target genes that might contribute to the cancer progression. To investigate the numbers of genes, lncRNAs, ciRNAs, and small molecules targeted by the top ranked miRNAs, miRNA-gene target interaction networks were constructed. A miRNA network showed some key molecules that were connected to top ranked miRNAs which might act as underlying drivers of survival in BLC. In conclusion, the identified miRNA signature would guide the understanding of the survival associated miRNAs and help develop miRNA target-based therapeutic strategies in BLC.

## Material and methods

### Dataset

#### The clinical characteristics

The miRNA expression profiles of 409 patients along with their survival times were retrieved from the TCGA database. All the data extraction methods were carried out in accordance with the TCGA guidelines and regulations. The patient selection criteria included patients with survival times and miRNA expression profiles. The miRNA expression was considered if the expression levels of mature miRNAs were presented in more than 70% of the samples. After the filtration process, there were 106 patients with miRNA expression profiles where each miRNA profile consisted of 485 miRNAs in the final dataset.

The clinical characteristics of the 106 patients with BLC are presented in Supplementary Fig. S11. The majority of BLC patients were male, and 69% were male and 31% female. The average age at diagnosis was 70.46 ± 9.43 and average height of the patients was 173.4 ± 11.14 cms. The total numbers of patients in stages 2, 3, and 4 were 14, 37, and 55, respectively. The range of survival times of patients were between 0.63 and 94.26 months.

### BLC-SVR method

BLC-SVR was designed to identify a set of miRNAs as a signature that could estimate the survival time in patients with BLC. BLC-SVR method was developed based on SVR incorporated with the optimal feature selection algorithm IBCGA. Two main parts of BLC-SVR are feature selection and survival estimation. BLC-SVR adopted the optimization technique from our previous study^29^.

### Feature selection algorithm IBCGA

BLC-SVR utilized the optimal feature selection algorithm IBCGA to select a minimum number of features from a large number of candidate features (miRNAs) while maximizing the prediction performance^32^. The IBCGA uses an intelligent evolutionary algorithm to solve the large parameter combinatorial optimization problems^64^. Here, we used genetic algorithm (GA) terms GA-chromosomes and GA-genes for the feature representation. The chromosome of IBCGA comprises 485 GA-genes and three 4-bit GA-genes to encode parameters *C*, γ, and ν for ν-SVR. The encoded GA-chromosomes were designed as described in previous studies^29–31^. The best prediction model of BLC-SVR was generated from the 50 independent runs of IBCGA. The main steps in IBCGA are described as follows: where the detailed description can be refer to the work^32^:

- Step 1: (Initialization) Randomly generate an initial population of individuals.
- Step 2: (Evaluation) Evaluate the fitness value of all individuals using the fitness function, which is to maximize the prediction accuracy (R^2^) in terms of 10-CV.
- Step 3: (Selection) Use a conventional method of tournament selection that selects a winner from two randomly selected individuals to generate a mating pool.
- Step 4: (Crossover) Select two parents from the mating pool to perform an orthogonal array crossover operation.
- Step 5: (Mutation) Apply a conventional mutation operator to the randomly selected individuals in the new population.
- Step 6: (Termination test) If the stopping condition for obtaining the solution is satisfied, output the best individual as the solution. Otherwise, go to Step 2.
- Step 7: (Inheritance) If *r* is less than a predefined number of features, randomly changes one bit in the binary GA-genes for each individual from 0 to 1, increase the number *r* by one and go to Step 2. Otherwise, stop the algorithm.

The applications of support vector machines (SVM) have diverse importance in biomedical sciences and precision medicine due to their capability in solving complications in predictions^65^. The SVM has two modules, support vector classifier (SVC) and support vector regression (SVR)^66^. The SVMs are used in various cancer diagnosis and prognosis predictions. The optimized SVMs were used to predict the cancer stage in breast cancer and hepatocellular carcinoma^26,27^, and estimation of survival in patients with lung adenocarcinoma, glioblastoma, neuroblastoma, and ovarian cancers^28–31^. The LibSVM package^67^ was used to implement the BLC-SVR. The optimization technique of SVR can be written as follows:

1 $${\text{min}}\left[ {\left\{ {\frac{1}{2}w^{T} (\emptyset \left( {x_{i} } \right) + b) + C(\nu \varepsilon + \frac{1}{m}\sum\limits_{{i = 1}}^{m} {(\xi _{i} + \xi _{i}^{*} )} )} \right\}} \right]$$

where $0\le\upnu \le$ 1, ${\xi }_{i}$ ≥ 0, ${\xi }_{i}^{*}$ ≥ 0, (*x*_*1*_, *y*_*1*_)…(*x*_*m*_, *y*_*m*_) are the input data points, *C* is the regularization parameter, *ε* is an insensitive loss function, and *b* is a constant.

### Performance measures

We used squared correlation coefficient (*R*^*2*^) and mean absolute error (*MAE*) as the estimation measures to evaluate the prediction performance of BLC-SVR.

2 $$R = \frac{{\sum\nolimits_{{i = 1}}^{N} {\left( {y_{i} - \bar{y}} \right)(z_{i} - \bar{z})} }}{{\left\lfloor {\sum\nolimits_{{i = 1}}^{N} {\left( {y_{i} - \bar{y}} \right)^{2} } } \right\rfloor \left[ {\sum\nolimits_{{i = 1}}^{N} {\left( {z_{i} - \bar{z}} \right)^{2} } } \right]}}$$

where *y*_i_ and *z*_i_ are the actual and predicted survival times of the *i*th miRNA, respectively, $\overline{y }$ and $\overline{z }$ are the corresponding means, and *N* is the total number of BLC patients in the validation set. The mean absolute error (*MAE*) is also used for the evaluation of prediction performance, defined as follows:

3 $$MAE= \frac{1}{N}\sum_{i=1}^{N}\left|{z}_{i}-{y}_{i}\right|$$

### Appearance score ASC

The robust signatures among the 50 independent runs of BLC-SVR has the highest ASC obtained using the following procedure^29^.

- Step 1: Perform *Ns* independent runs of BLC-SVR for obtaining *Ns* miRNA signatures. There are *m*_*i*_ features in the *i*th signatures, *i* = 1, …, *Ns (in this study Ns* = *50)*.
- Step 2: The *ASC* of a miRNA signature is calculated as follows:
  - (1) Calculate the appearance frequency *f(miR)*for each feature *miR* that appears in the *Ns* signatures.
  - (2) Calculate the score *F*_*i*_, *i* = 1, …, *Ns.* Where *miR*_*it*_ is the *t*th feature in the *i*th signature:
    4 $${F}_{i}=\sum_{t=1}^{{m}_{i}}f\left({miR}_{it}\right)/{m}_{i}$$
  - (3) Obtain the *i*-th feature set with the highest appearance score *Fi* as the robust signature.

### Ridge regression, Lasso and elastic net

The estimation performance of BLC-SVR was compared with some standard regression methods, ridge regression, Lasso, and elastic net. Ridge regression is a penalized regression approach where the Euclidean norm was used as the penalty^68^. Lasso uses L1 regularization to identify features and regression coefficients by regularizing the coefficients to zero that lead to minimize the prediction error^69^. Elastic net is a combination of Lasso and ridge regression^70^. The minimum λ was chosen after 100 iterations of 10-CV for ridge, Lasso and elastic net. The prediction performance was evaluated in terms of the correlation coefficient and mean absolute error.

### KEGG pathway and GO annotation analysis

The DIANA-micro-T-CDS algorithm provided the predicted miRNA targets for the pathway analysis^71^. The p-value threshold was set to 0.05, and Fishers’s exact test (hypergeometric distribution) was used for the enrichment analysis.

### miRNA-interaction network

The miRNA-target interaction networks were built using miRNet 2.0: a miRNA-centric network visual analytics platform^49^. For better visualization of target genes, we reduced the less important edges based on the shortest path measures, where the number of edges within the network can be reduced significantly by keeping the shortest path between hub-nodes. We used short distance and minimum layout filters for lncRNA and ciRNA networks, respectively.

## Supplementary Information

Supplementary Information.

## Data Availability

All the data used in this analysis can be found on the TCGA data portal [https://portal.gdc.cancer.gov/].
