# Author Correction: MicroRNA signature for estimating the survival time in patients with bladder urothelial carcinoma

**DOI:** 10.1038/s41598-022-09235-4

**Published:** 2022-03-23

**Authors:** Srinivasulu Yerukala Sathipati, Ming‑Ju Tsai, Sanjay K. Shukla, Shinn‑Ying Ho, Yi Liu, Afshin Beheshti

**Affiliations:** 1 Center for Precision Medicine Research, Marshfield Clinic Research Institute, Marshfield, WI 54449 USA; 2 Hinda and Arthur Marcus Institute for Aging Research at Hebrew Senior Life, Boston, MA USA; 3 Department of Medicine, Beth Israel Deaconess Medical Center and Harvard Medical School, Boston, MA USA; 4 Institute of Bioinformatics and Systems Biology, National Yang Ming Chiao Tung University, Hsinchu, Taiwan; 5 College of Health Sciences, Kaohsiung Medical University, Kaohsiung, Taiwan; 6 Biomedical Engineering, National Yang Ming Chiao Tung University, Hsinchu, Taiwan; 7 KBR, Space Biosciences Division, NASA Ames Research Center, Moffett Field, CA 94035 USA; 8 Stanley Center for Psychiatric Research, Broad Institute of MIT and Harvard, Cambridge, MA 02142 USA

Correction to: *Scientific Reports* 10.1038/s41598-022-08082-7, published online 09 March 2022

The original version of this Article contained an error in the spelling of the author Afshin Beheshti, which was incorrectly given as Afshin Behesthi.

This error has now been corrected in the original Article and in the accompanying Supplementary Information file.

