# Supplementary material for: MicroRNA signature for estimating the survival time in patients with bladder urothelial carcinoma
Source: Sci Rep. 2022 Mar 9;12:4141. doi: 10.1038/s41598-022-08082-7 (PMC8907292; doi:10.1038/s41598-022-08082-7)
Supplement: Supplementary file 1 — Supplementary Information. [file 41598_2022_8082_MOESM1_ESM.docx]

# Supplementary information

# MicroRNA signatures for estimating the survival time in patients with bladder urothelial carcinoma

**Srinivasulu Yerukala Sathipati^1*^, Ming-Ju Tsai^2,3^, Sanjay K Shukla^1^, Shinn-Ying Ho^4,5^, Yi Liu^6^ and Afshin Beheshti^7,8^**

^1^Center for Precision Medicine Research, Marshfield Clinic Research Institute, Marshfield, WI, 54449, USA

^2^Hinda and Arthur Marcus Institute for Aging Research at Hebrew Senior Life, Boston, MA, USA

^3^Department of Medicine, Beth Israel Deaconess Medical Center and Harvard Medical School, Boston, MA, USA

^4^Institute of Bioinformatics and Systems biology, National Yang Ming Chiao Tung University, Hsinchu, Taiwan

^5^College of Health Sciences, Kaohsiung Medical University, Kaohsiung, Taiwan

^6^Biomedical Engineering, National Yang Ming Chiao Tung University, Hsinchu, Taiwan

^7^KBR, Space Biosciences Division, NASA Ames Research Center, Moffett Field, CA, 94035, USA

^8^Stanley Center for Psychiatric Research, Broad Institute of MIT and Harvard, Cambridge, MA, 02142, USA

# *Corresponding author: Email address: [sathipathi.srinivasulu@marshfieldclinic.org](mailto:sathipathi.srinivasulu@marshfieldclinic.org)

# Supplementary Figures

**Supplementary Figure S1**. Appearance score measurement for prediction models of BLC-SVR. The highest Appearance score obtained is 17.27.


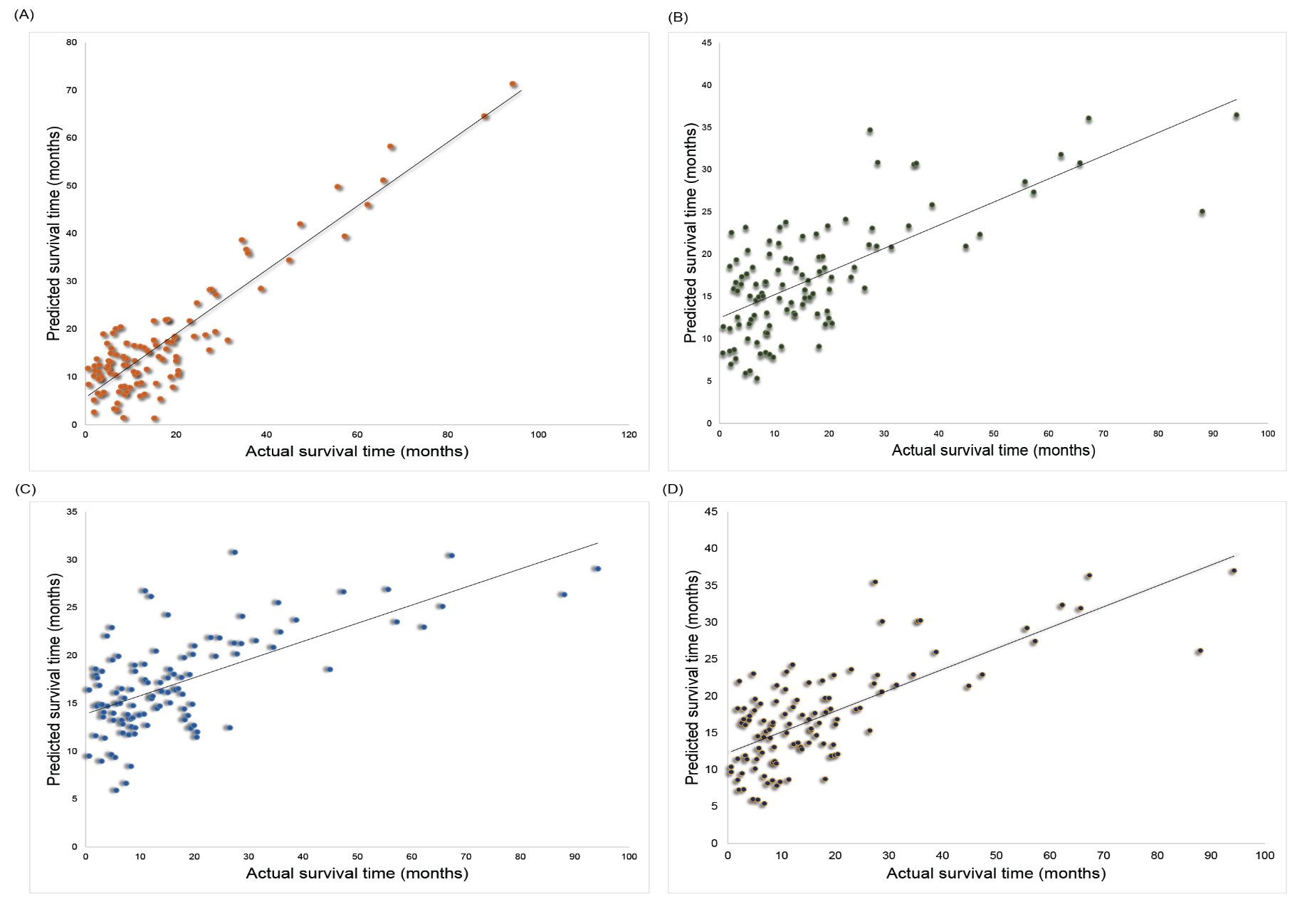


**Supplementary Figure S2**. The comparison of estimation performance. (A) Estimation performance of BLC-SVR (R^2^=0.83), (B) Lasso (R^2^=0.50), (C) ridge regression (R^2^=0.42), and (D) elastic net (R^2^=0.52).


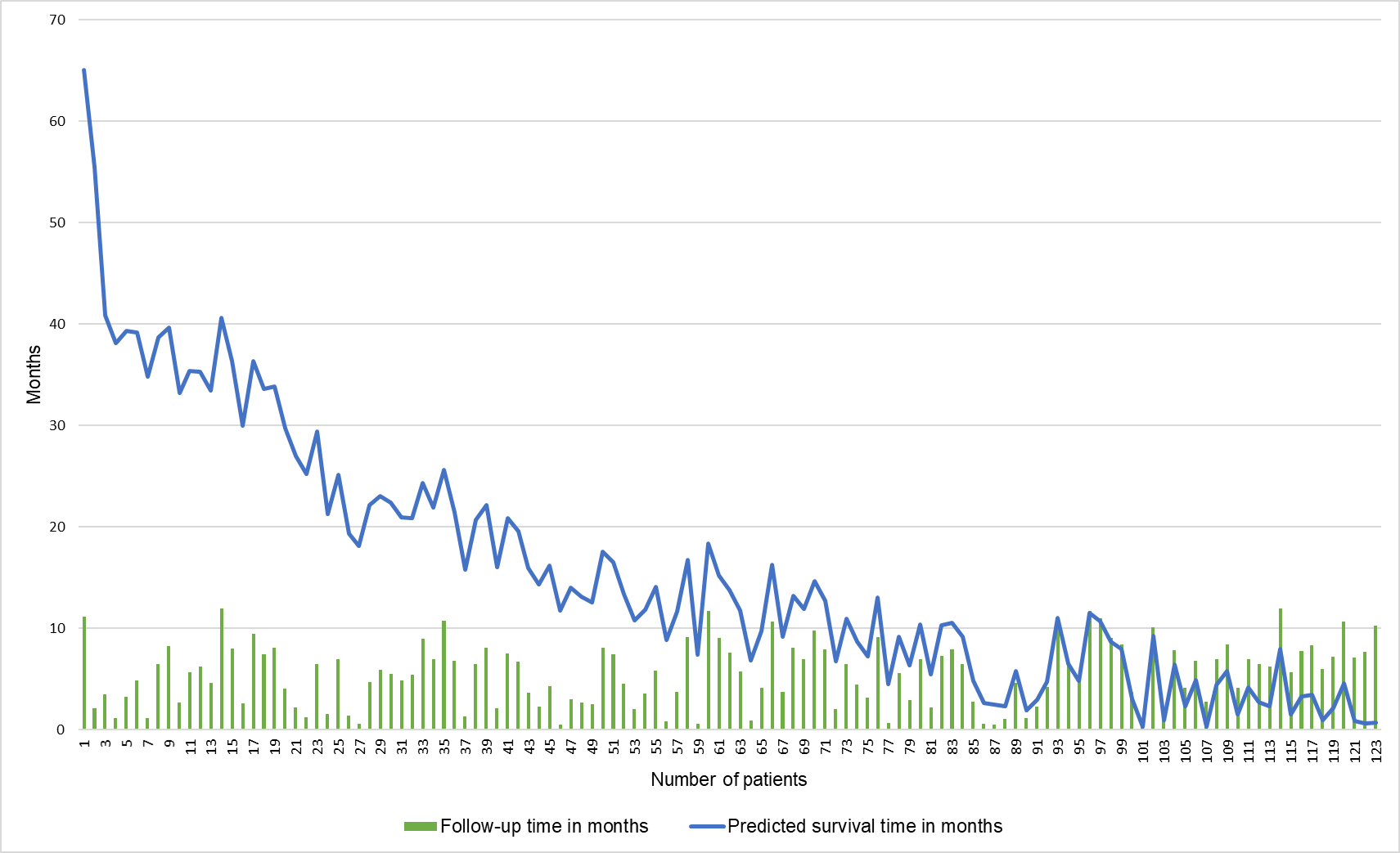


**Supplementary Figure S3**. The estimation performance of BLC-SVR on an independent test cohort consisting of 123 patients with bladder cancer.


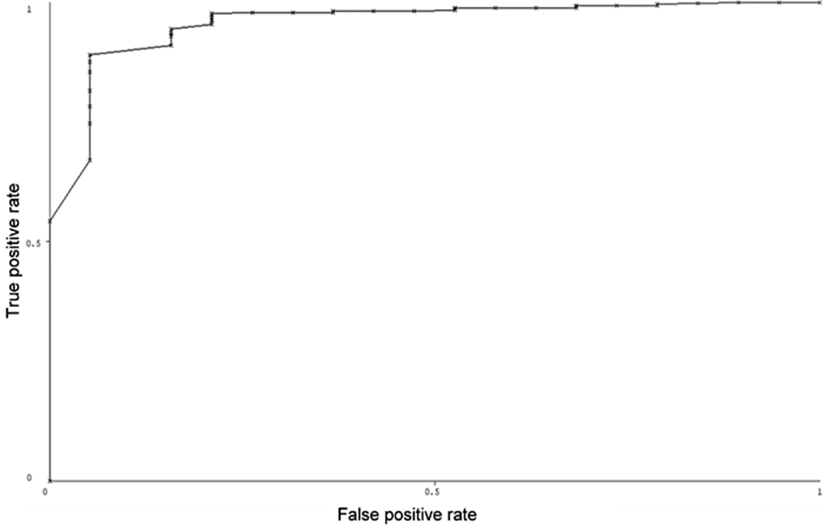


**Supplementary Figure S4**. The diagnostic ability of top 10 ranked miRNAs evaluated using ROC curve (AUC = 0.95).


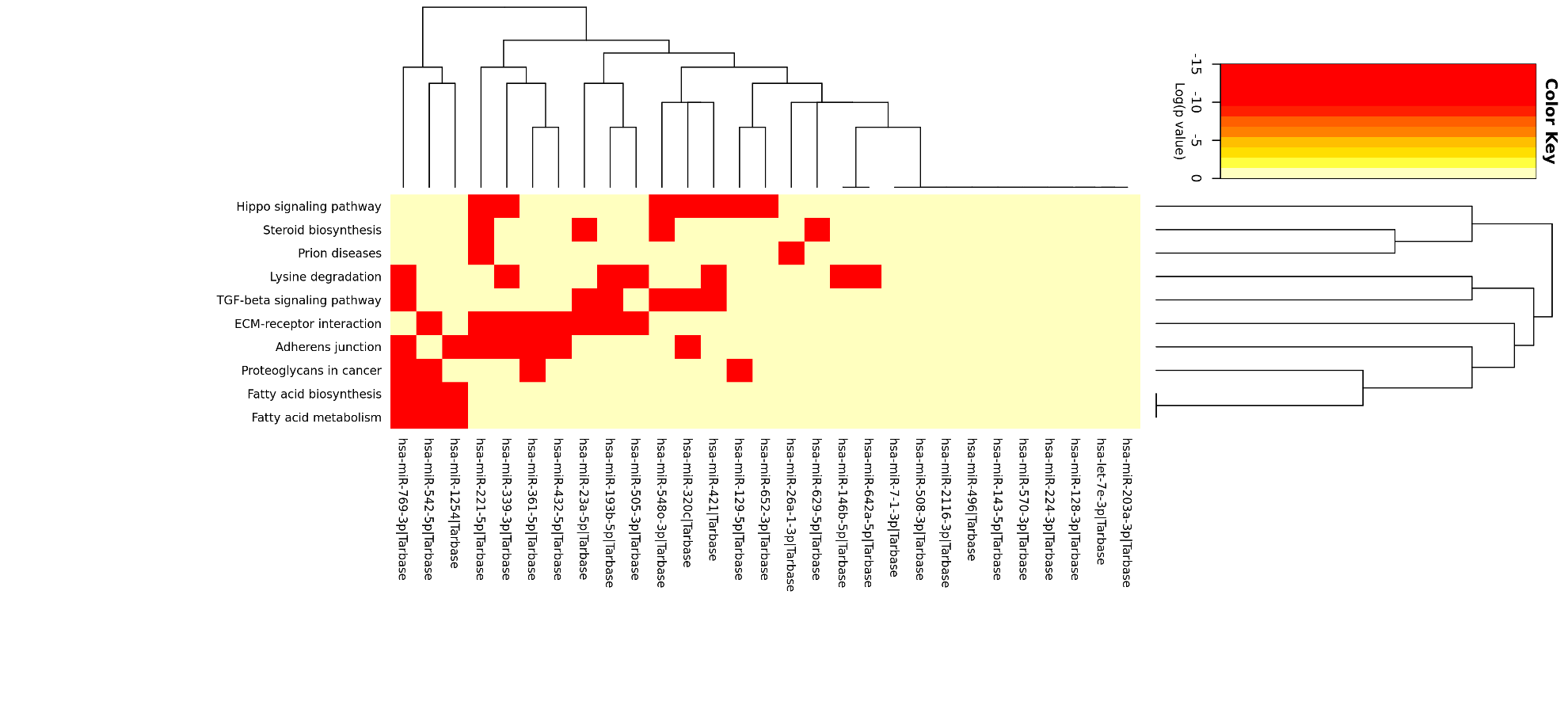


**Supplementary Figure S5**. The identified miRNA signature enriched in various KEGG pathways.


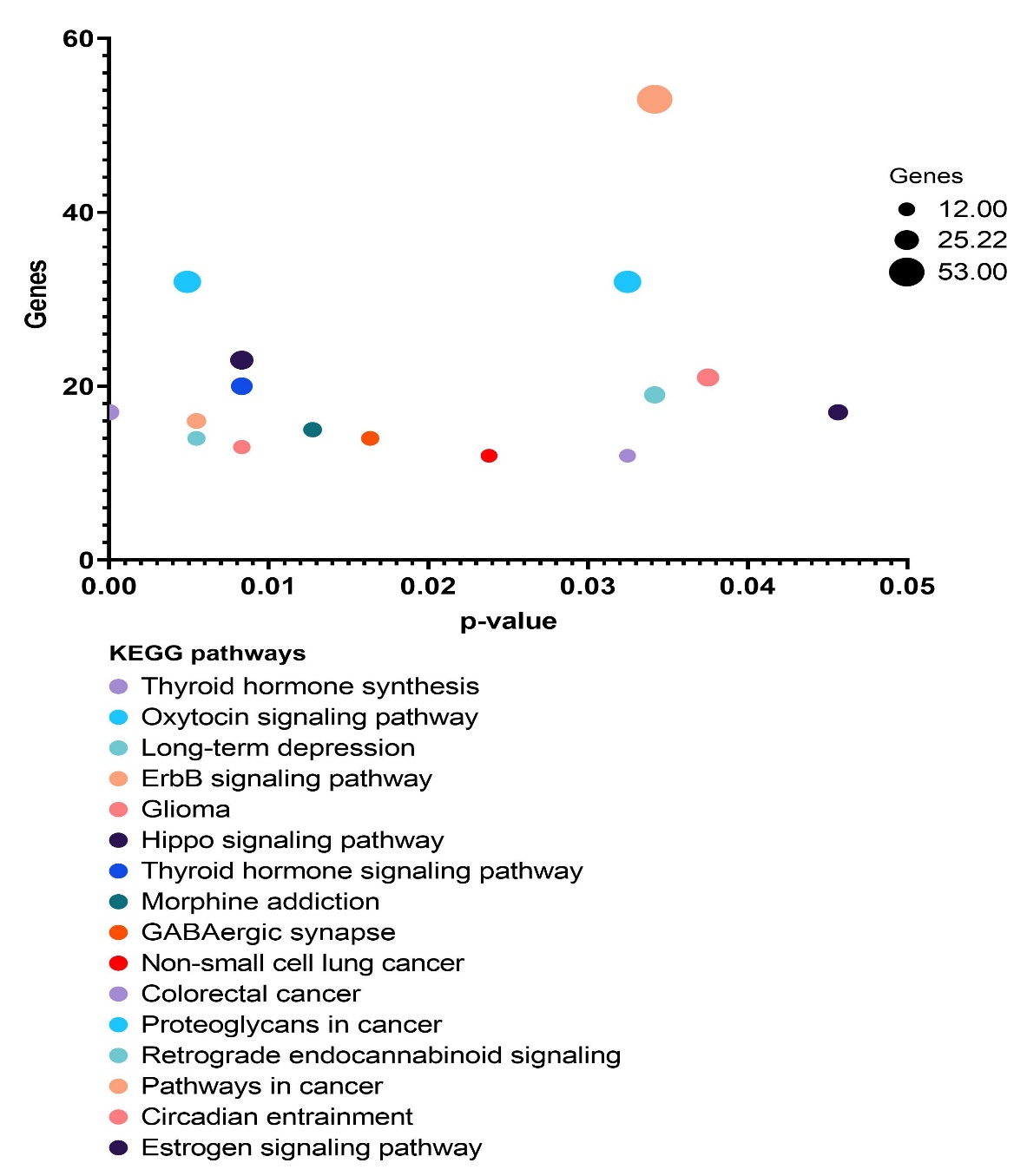


**Supplementary Figure S6**. The identified KEGG pathways in bladder cancer stage II and III.


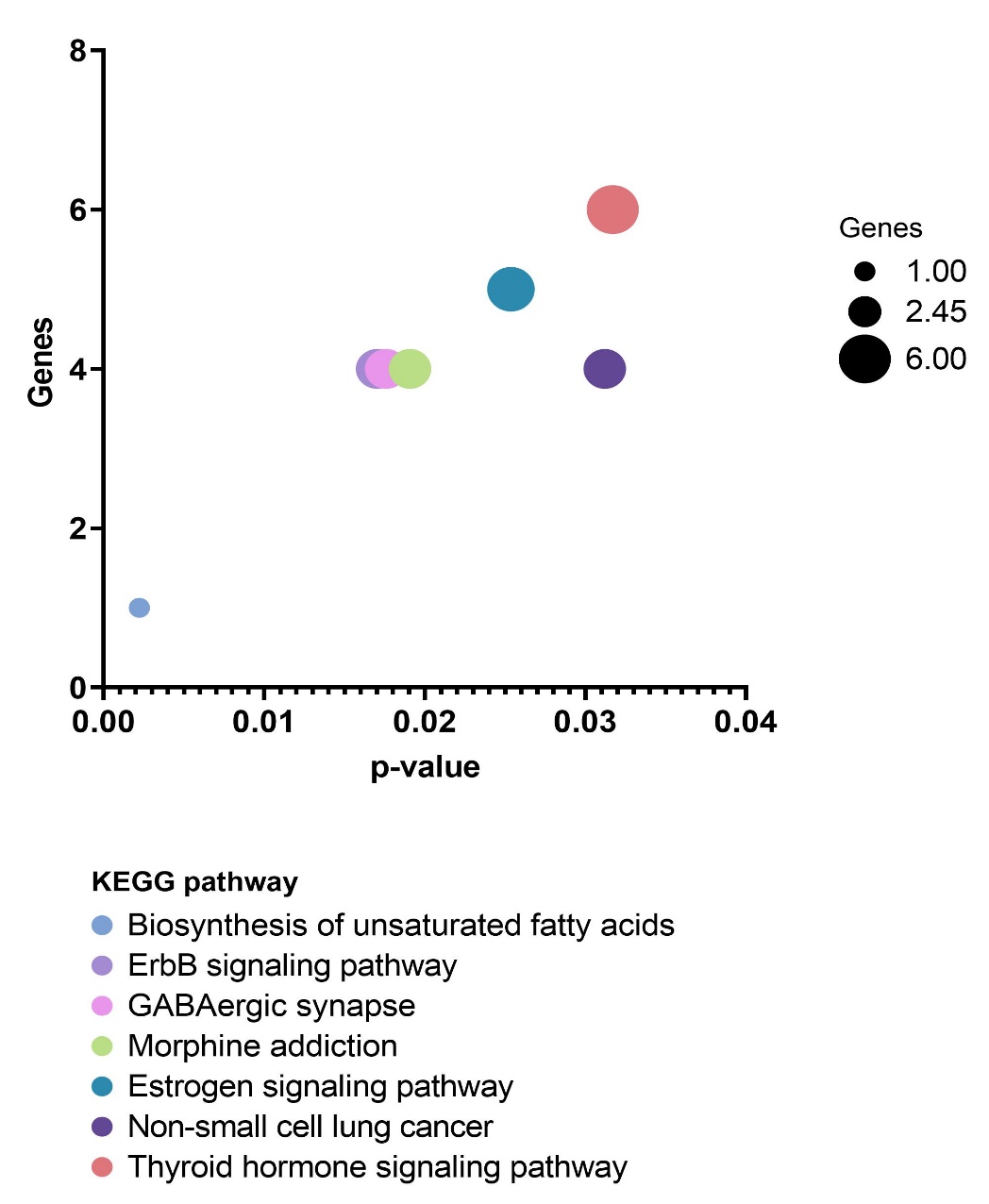


**Supplementary Figure S7**. The identified KEGG pathways in bladder cancer stage III and IV.


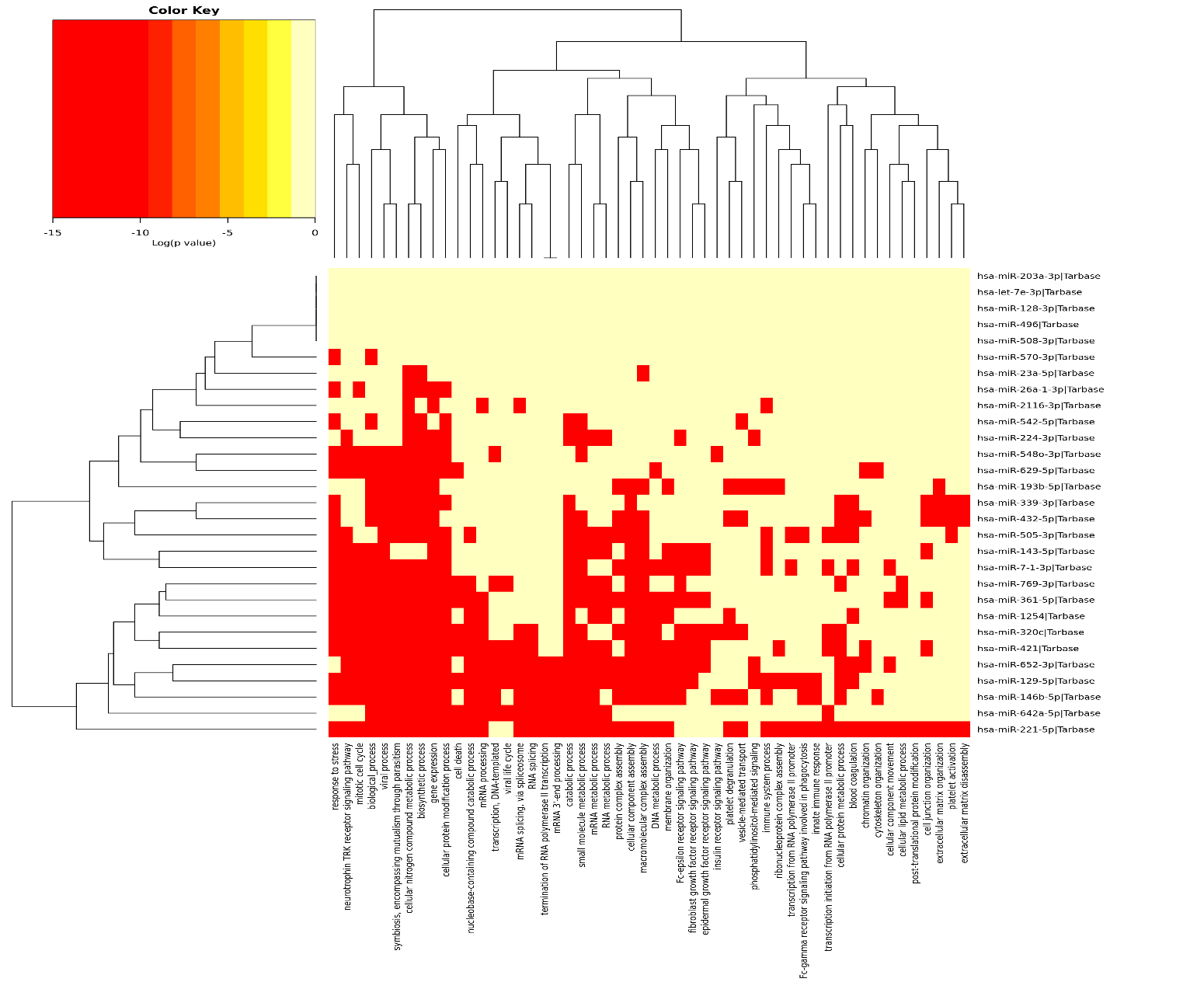


**Supplementary Figure S8**. The GO annotation analysis of the identified miRNA signature enriched in various biological process


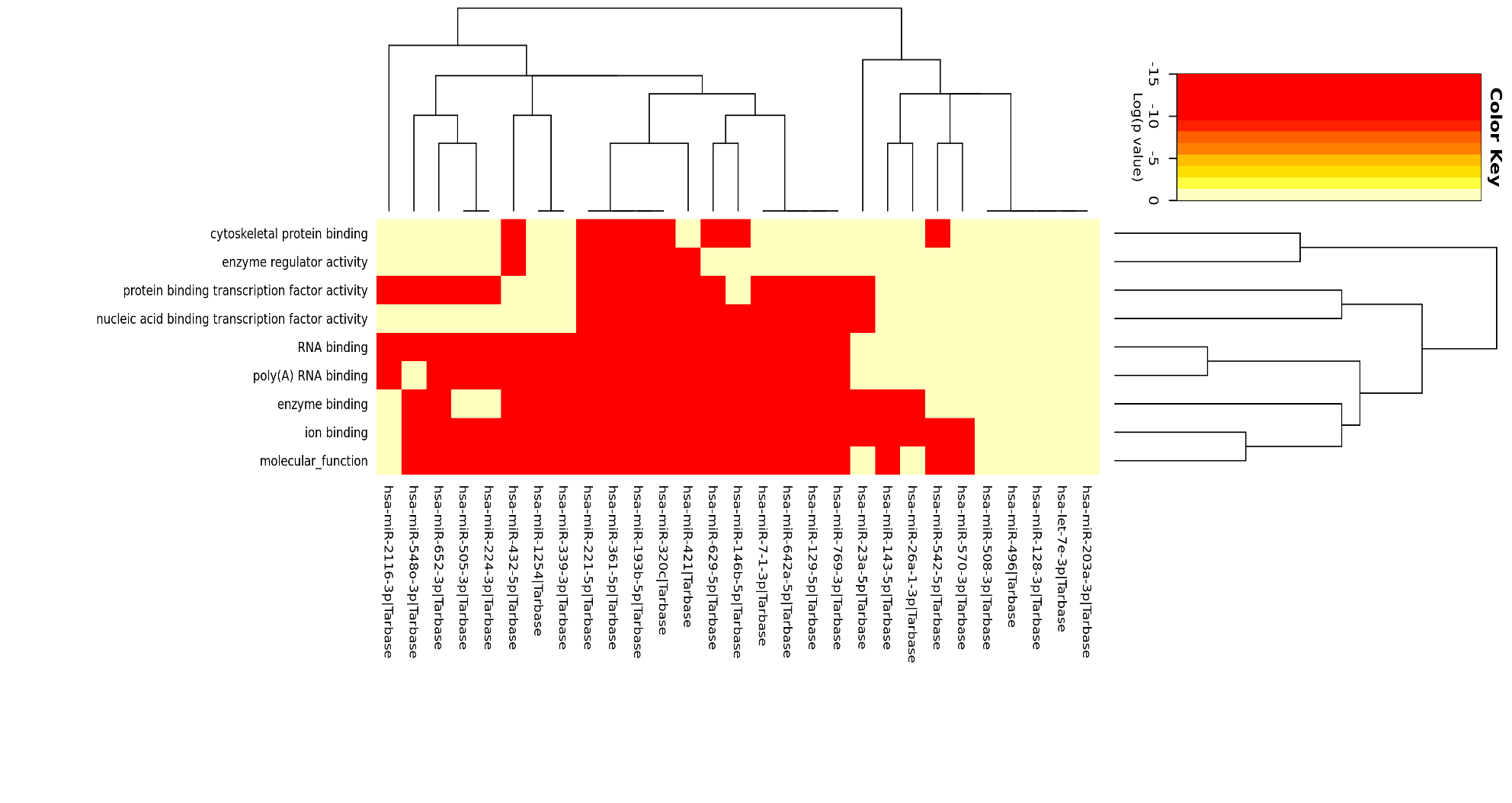


**Supplementary Figure S9**. The GO annotation analysis of the identified miRNA signature enriched in various molecular functions.


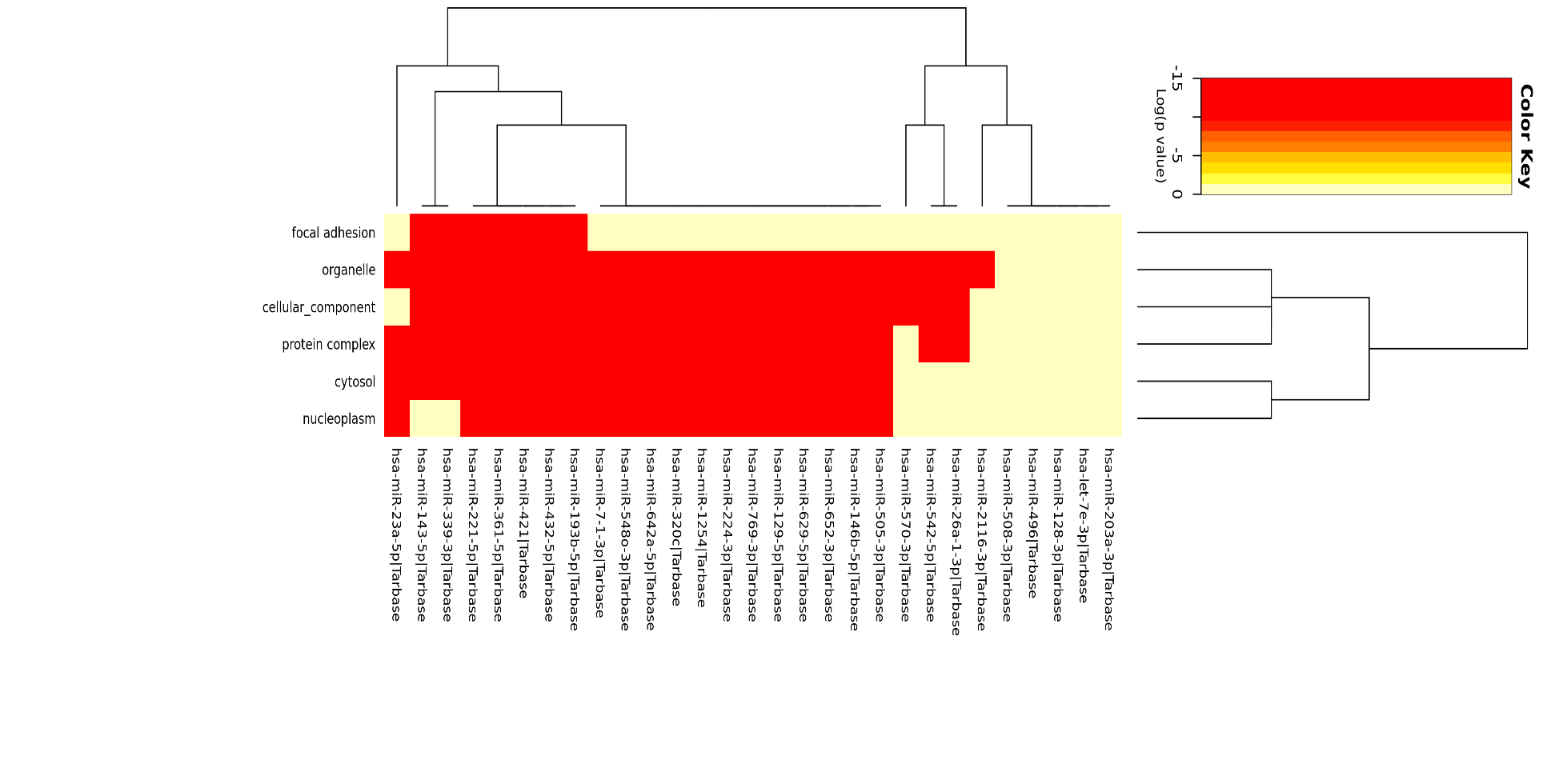


**Supplementary Figure S10**. The GO annotation analysis of the identified miRNA signature enriched in various cellular components.


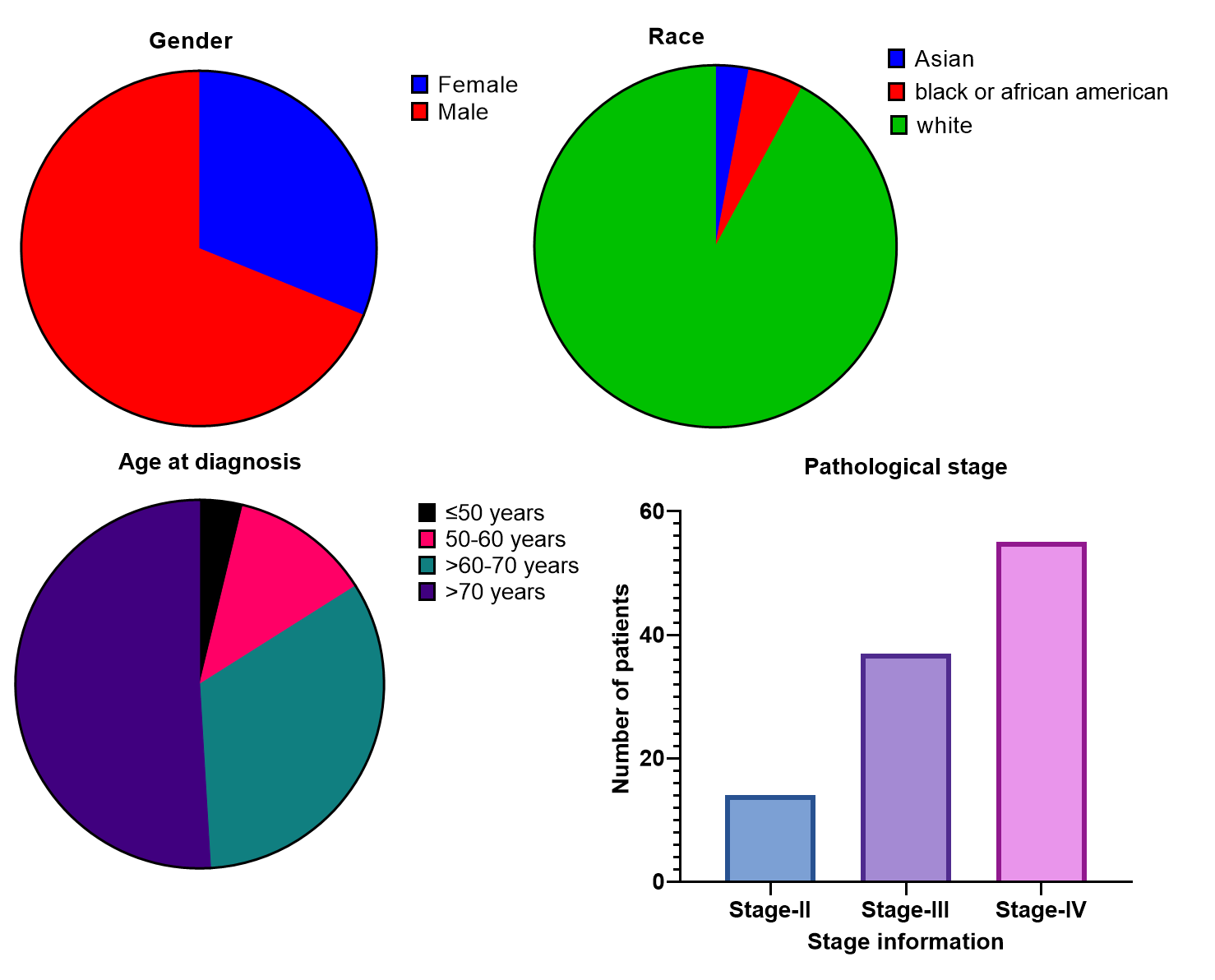


**Supplementary Figure S11**. Clinical characteristics of bladder cancer cohort.

# Supplementary Tables

**Supplementary Table S1**. The KEGG pathway analysis of the miRNA signature.

| **KEGG pathways** | **p-value** | **Genes** | **miRNAs** |
| --- | --- | --- | --- |
| Prion diseases | <0.0001 | 5 | 2 |
| Fatty acid biosynthesis | <0.0001 | 1 | 3 |
| Fatty acid metabolism | 2.24E-09 | 5 | 3 |
| ECM-receptor interaction | 2.24E-09 | 27 | 8 |
| Hippo signaling pathway | 2.24E-09 | 44 | 7 |
| Adherens junction | 2.90E-07 | 31 | 7 |
| Steroid biosynthesis | 2.23E-06 | 4 | 4 |
| Lysine degradation | 0.000171 | 16 | 7 |
| TGF-beta signaling pathway | 0.000536 | 15 | 6 |
| Proteoglycans in cancer | 0.001492 | 40 | 4 |

**Supplementary Table** **S2**. The KEGG pathway analysis of miRNAs across different stages of bladder cancer.

| **Cancer stage** | **KEGG pathway** | **p-value** | **Genes** | **MiRNAs** |
| --- | --- | --- | --- | --- |
| Stage II&III | Thyroid hormone synthesis | 9.29E-07 | 17 | 5 |
|  | Oxytocin signaling pathway | 0.004889 | 32 | 8 |
|  | Long-term depression | 0.005463 | 14 | 5 |
|  | ErbB signaling pathway | 0.005463 | 16 | 6 |
|  | Glioma | 0.008303 | 13 | 5 |
|  | Hippo signaling pathway | 0.008303 | 23 | 6 |
|  | Thyroid hormone signaling pathway | 0.008303 | 20 | 6 |
|  | Morphine addiction | 0.01274 | 15 | 5 |
|  | GABAergic synapse | 0.016345 | 14 | 5 |
|  | Non-small cell lung cancer | 0.02379 | 12 | 5 |
|  | Colorectal cancer | 0.032464 | 12 | 5 |
|  | Proteoglycans in cancer | 0.032464 | 32 | 6 |
|  | Retrograde endocannabinoid signaling | 0.034165 | 19 | 6 |
|  | Pathways in cancer | 0.034165 | 53 | 7 |
|  | Circadian entrainment | 0.037508 | 21 | 5 |
|  | Estrogen signaling pathway | 0.045654 | 17 | 5 |
| Stage III&IV | Biosynthesis of unsaturated fatty acids | 2.16E-05 | 1 | 1 |
|  | ErbB signaling pathway | 0.017018 | 4 | 1 |
|  | GABAergic synapse | 0.017018 | 4 | 1 |
|  | Morphine addiction | 0.019068 | 4 | 1 |
|  | Estrogen signaling pathway | 0.025357 | 5 | 1 |
|  | Non-small cell lung cancer | 0.0312 | 4 | 1 |
|  | Thyroid hormone signaling pathway | 0.031695 | 6 | 1 |
|  | Fatty acid metabolism | 0.041271 | 1 | 1 |
|  | Central carbon metabolism in cancer | 0.041271 | 5 | 1 |
|  | Ovarian steroidogenesis | 0.044827 | 3 | 1 |

**Supplementary Table S3.** The miRNA signature involvement in biological processes.

| **GO Category** | **p-value** | **Genes** | **miRNAs** |
| --- | --- | --- | --- |
| DNA metabolic process | <1e-325 | 209 | 10 |
| cellular protein metabolic process | <1e-325 | 138 | 10 |
| membrane organization | <1e-325 | 183 | 10 |
| RNA metabolic process | <1e-325 | 95 | 11 |
| nucleobase-containing compound catabolic process | <1e-325 | 253 | 11 |
| mRNA metabolic process | <1e-325 | 91 | 12 |
| mitotic cell cycle | <1e-325 | 161 | 14 |
| cellular component assembly | <1e-325 | 390 | 15 |
| neurotrophin TRK receptor signaling pathway | <1e-325 | 102 | 15 |
| macromolecular complex assembly | <1e-325 | 269 | 15 |
| small molecule metabolic process | <1e-325 | 614 | 16 |
| catabolic process | <1e-325 | 570 | 17 |
| symbiosis, encompassing mutualism through parasitism | <1e-325 | 253 | 17 |
| response to stress | <1e-325 | 622 | 18 |
| viral process | <1e-325 | 231 | 18 |
| cellular protein modification process | <1e-325 | 704 | 19 |
| biosynthetic process | <1e-325 | 1181 | 21 |
| gene expression | <1e-325 | 320 | 21 |
| cellular nitrogen compound metabolic process | <1e-325 | 1509 | 22 |
| cell death | 9.21E-15 | 214 | 8 |
| mRNA processing | 1.20E-14 | 141 | 10 |
| Fc-epsilon receptor signaling pathway | 1.21E-14 | 46 | 10 |
| RNA splicing | 1.48E-14 | 89 | 7 |
| protein complex assembly | 1.29E-13 | 195 | 10 |
| blood coagulation | 1.45E-13 | 99 | 8 |
| mRNA splicing, via spliceosome | 8.73E-12 | 66 | 8 |
| immune system process | 3.14E-10 | 249 | 8 |
| transcription, DNA-templated | 1.30E-08 | 356 | 7 |
| platelet degranulation | 2.75E-08 | 25 | 6 |
| epidermal growth factor receptor signaling pathway | 1.43E-07 | 47 | 6 |
| cell junction organization | 1.61E-06 | 47 | 6 |
| platelet activation | 6.38E-06 | 36 | 4 |
| fibroblast growth factor receptor signaling pathway | 7.87E-06 | 44 | 7 |
| transcription initiation from RNA polymerase II promoter | 9.17E-06 | 53 | 6 |
| vesicle-mediated transport | 1.05E-05 | 159 | 6 |
| chromatin organization | 1.30E-05 | 39 | 5 |
| viral life cycle | 3.99E-05 | 29 | 5 |
| termination of RNA polymerase II transcription | 9.21E-05 | 17 | 5 |
| activation of signaling protein activity involved in unfolded protein response | 0.000517 | 23 | 4 |
| extracellular matrix organization | 0.000558 | 54 | 4 |
| ribonucleoprotein complex assembly | 0.000691 | 34 | 4 |

**Supplementary Table S4.** The miRNA signature involvement in molecular functions

| **GO Category** | **p-value** | **Genes** | **miRNAs** |
| --- | --- | --- | --- |
| ion binding | 7.14E-146 | 2390 | 29 |
| nucleic acid binding transcription factor activity | 2.55E-41 | 445 | 26 |
| protein binding transcription factor activity | 1.50E-32 | 235 | 27 |
| enzyme binding | 6.13E-30 | 516 | 28 |
| enzyme regulator activity | 1.90E-11 | 304 | 27 |
| cytoskeletal protein binding | 1.07E-10 | 279 | 26 |
| RNA binding | 6.62E-07 | 617 | 28 |
| small conjugating protein binding | 0.000403 | 41 | 18 |
| histone binding | 0.003955 | 76 | 25 |
| protein binding, bridging | 0.00557 | 60 | 24 |
| transmembrane transporter activity | 0.010082 | 332 | 28 |

**Supplementary Table S5**. The miRNA signature involvement in cellular components.

| **GO Category** | **p-value** | **Genes** | **miRNAs** |
| --- | --- | --- | --- |
| nucleoplasm | <1e-325 | 407 | 8 |
| protein complex | <1e-325 | 995 | 8 |
| protein binding transcription factor activity | <1e-325 | 203 | 10 |
| epidermal growth factor receptor signaling pathway | <1e-325 | 103 | 10 |
| gene expression | <1e-325 | 220 | 10 |
| nucleic acid binding transcription factor activity | <1e-325 | 385 | 11 |
| Fc-epsilon receptor signaling pathway | <1e-325 | 87 | 11 |
| enzyme binding | <1e-325 | 457 | 12 |
| neurotrophin TRK receptor signaling pathway | <1e-325 | 135 | 12 |
| cellular protein modification process | <1e-325 | 886 | 15 |
| ion binding | <1e-325 | 2222 | 16 |
| biosynthetic process | <1e-325 | 1454 | 17 |
| cellular nitrogen compound metabolic process | <1e-325 | 1802 | 20 |
| organelle | <1e-325 | 3748 | 23 |
| transcription, DNA-templated | 7.44E-15 | 682 | 8 |
| cellular component assembly | 9.33E-15 | 342 | 7 |
| cytosol | 1.14E-14 | 638 | 6 |
| symbiosis, encompassing mutualism through parasitism | 2.05E-14 | 165 | 8 |
| fibroblast growth factor receptor signaling pathway | 1.4E-13 | 84 | 7 |
| blood coagulation | 1.45E-12 | 157 | 7 |
| viral process | 1.69E-11 | 140 | 7 |
| response to stress | 1.65E-09 | 592 | 8 |
| phosphatidylinositol-mediated signaling | 8.25E-09 | 58 | 8 |
| synaptic transmission | 1.38E-08 | 125 | 5 |
| cell death | 2.8E-08 | 213 | 5 |
| mitotic cell cycle | 5.97E-08 | 96 | 5 |
| macromolecular complex assembly | 6.35E-07 | 211 | 7 |
| catabolic process | 2.26E-06 | 474 | 7 |
| small molecule metabolic process | 1.24E-05 | 483 | 6 |
| nervous system development | 8.49E-05 | 106 | 3 |
| transcription initiation from RNA polymerase II promoter | 0.0002 | 64 | 5 |
